# Beyond Neuropathy: The Mechanisms and Phenotypes of Diabetes-Related Musculoskeletal Pain

**DOI:** 10.3390/jcm15072639

**Published:** 2026-03-31

**Authors:** Lívia Bernardi Nardini, Julia Kortstee Ferreira, Jo Nijs, Isabel C. N. Sacco, Paula Rezende Camargo

**Affiliations:** 1 Laboratory of Analysis and Intervention of the Shoulder Complex, Department of Physical Therapy, Universidade Federal de São Carlos, São Carlos 13565-905, Brazil; livia.b.nardini@gmail.com (L.B.N.); juliakortstee@gmail.com (J.K.F.); prcamargo@ufscar.br (P.R.C.); 2 Pain in Motion Research Group, Department of Physiotherapy, Human Physiology and Anatomy, Faculty of Physical Education and Physiotherapy, Vrije Universiteit Brussel, 1050 Brussels, Belgium; 3 Pijnpraxis.be Interdisciplinary Practice for Pain Management, 3970 Leopoldsburg, Belgium; 4 Department of Health and Rehabilitation, Unit of Physiotherapy, Institute of Neuroscience and Physiology, Sahlgrenska Academy, University of Gothenburg, 405 30 Göteborg, Sweden; 5 Physical Therapy, Speech and Occupational Therapy Department, School of Medicine, University of São Paulo, São Paulo 01246-903, Brazil; icnsacco@usp.br

**Keywords:** diabetes mellitus, neuropathic pain, nociceptive pain, nociplastic pain, pain phenotyping, precision medicine

## Abstract

Diabetes Mellitus (DM) is a major global health concern associated with serious complications, high healthcare costs, and reduced quality of life. Musculoskeletal pain is a common complication and contributes to limitations in daily activities and increased healthcare utilization. Pain is a multidimensional phenomenon typically classified as nociceptive, neuropathic, or nociplastic; however, evidence regarding pain mechanisms and phenotypes in people with DM remains limited. This review aimed to synthesize and critically review the literature on musculoskeletal pain in individuals with DM, focusing on pain mechanisms and phenotypes according to the International Association for the Study of Pain (IASP) classification. A narrative review with systematic search procedures examined the applicability of the seven clinical criteria proposed by the IASP for nociplastic pain to musculoskeletal pain phenotyping. Searches were conducted in PubMed and Web of Science from inception to December 2025. Evidence was analyzed according to the IASP nociplastic pain criteria to explore alignment with pain phenotyping approaches. Overall, the literature indicates that neuropathic pain mechanisms are relatively well documented and consistently reported in individuals with diabetes, while nociceptive musculoskeletal drivers are also described but with more limited and heterogeneous evidence. In contrast, evidence addressing hypersensitivity phenomena and other domains related to nociplastic pain remains scarce and is still emerging. This pattern suggests that current research on pain in people with DM remains focused on neuropathic mechanisms. Future research adopting standardized pain phenotyping frameworks is needed to support more precise and individualized pain management strategies in this population.

## 1. Introduction

Diabetes Mellitus (DM) is a highly prevalent metabolic disorder affecting more than half a billion people worldwide [1]. Chronic hyperglycemia can lead to serious systemic complications [2], which are expensive to treat and negatively impact quality of life [3]. In addition to these complications, pain is frequently reported by individuals with DM, particularly musculoskeletal pain, which appears to be more common in this population than in the general population [4,5,6]. Despite this, studies on pain in people with DM have focused mainly on specific diagnoses, such as diabetic neuropathy and frozen shoulder, while the underlying mechanisms contributing to pain have received comparatively less attention.

Pain is defined by the International Association for the Study of Pain (IASP) as an unpleasant sensory and emotional experience associated with, or resembling, actual or potential tissue damage [7]. The high prevalence of pain and pain-related conditions contributes substantially to disability worldwide [8]. Nociceptive, neuropathic and nociplastic pain are the three main pain phenotypes described by the IASP, with nociplastic pain being introduced as a third mechanistic descriptor only in 2017 [9,10]. Although each phenotype presents distinct definitions and key features [11], it is increasingly recognized that many individuals experience pain states in which more than one mechanism is present [10].

The literature investigating pain symptoms in people with DM has increased in recent years [6,11,12,13,14]. However, most studies do not explore the different pain phenotypes according to the IASP clinical criteria, potentially overlooking a broader understanding of pain in this population. Diabetic neuropathy is a common complication of DM and frequently presents with pain symptoms, such as tingling, pricking and numbness [15,16,17,18,19,20]. In practice, the literature often focuses only on the neuropathic domain of these symptoms [15,21,22,23]. Nociplastic pain is a relatively recent concept and has not yet been investigated in people with DM. It refers to pain that arises from altered nociceptive processing in the absence of clear evidence of ongoing tissue damage or a somatosensory lesion sufficient to explain the pain [24]. Importantly, this does not imply the absence of peripheral pathology, but rather that the observed pathology may be insufficient to fully account for the pain experience [24]. In this context, persistent nociceptive input may contribute to the development of nociplastic pain, as hypersensitivity has been linked to longer duration of nociceptive pain [25,26] and increased rates of nociplastic pain states [27,28].

Precision medicine refers to an evidence-based approach that aims to tailor treatment strategies by identifying clinically meaningful patient subgroups based on characteristics such as disease mechanisms, prognosis, or treatment response [29]. In the context of pain, this perspective has been operationalized through the identification of pain phenotypes, such as nociceptive, neuropathic, and nociplastic pain, which involve distinct biological mechanisms and may respond differently to treatment. This approach has been explored in populations such as cancer survivors [11], COVID-19 survivors [30], and individuals with low back pain [31], where the identification of predominant pain mechanisms has been proposed to support more mechanism-informed pain management. However, in individuals with DM, the assessment and management of pain often follow a more generalized approach, with limited consideration of the predominant pain mechanisms. Recognizing pain phenotypes is particularly important in this context, as neuropathic and nociplastic pain tend to be more difficult to manage than nociceptive pain. Consequently, treatments that are effective for nociceptive pain may be less effective—or even worsen symptoms—in individuals with other pain phenotypes, particularly nociplastic pain [30].

In this context, this narrative review aimed to synthesize and critically interpret the existing literature on musculoskeletal pain in individuals with DM, with a focus on pain mechanisms and phenotypes, as well as identifying current evidence and knowledge gaps. To guide the overall interpretation of the evidence, a conceptual framework was developed.

## 2. Methods

### 2.1. Study Design

This study is a narrative review with systematic search procedures. This approach allowed the integration and critical interpretation of the literature to explore the applicability of the IASP nociplastic pain criteria to musculoskeletal pain in individuals with DM.

### 2.2. Conceptual Framework

**Figure 1:**
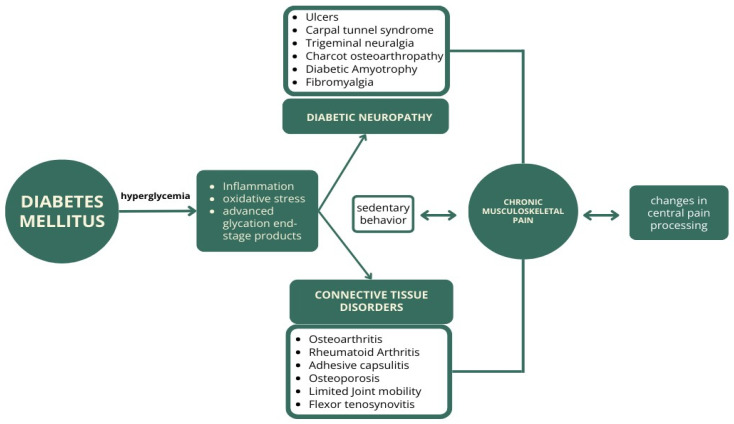
Conceptual framework illustrating potential pathways linking DM to chronic musculoskeletal pain, including metabolic alterations (e.g., inflammation, oxidative stress, and advanced glycation end-products), and their potential contributions to neuropathic and musculoskeletal complications, as well as interactions with central pain processing.

We developed a conceptual framework primarily based on literature reviews on the topic [32,33,34,35,36] to illustrate potential pathways linking DM to chronic musculoskeletal pain (Figure 1). The framework highlights hyperglycemia-related metabolic alterations, including inflammation, oxidative stress, and the accumulation of advanced glycation end-products, which may lead to two main groups of complications: diabetic neuropathy and connective muscular tissue disorders. These pathways may contribute to musculoskeletal pain and may interact with sedentary behavior and changes in central pain processing. This framework guided the extraction and interpretation of evidence in this narrative review.

### 2.3. Search Strategy

**Figure 2:**
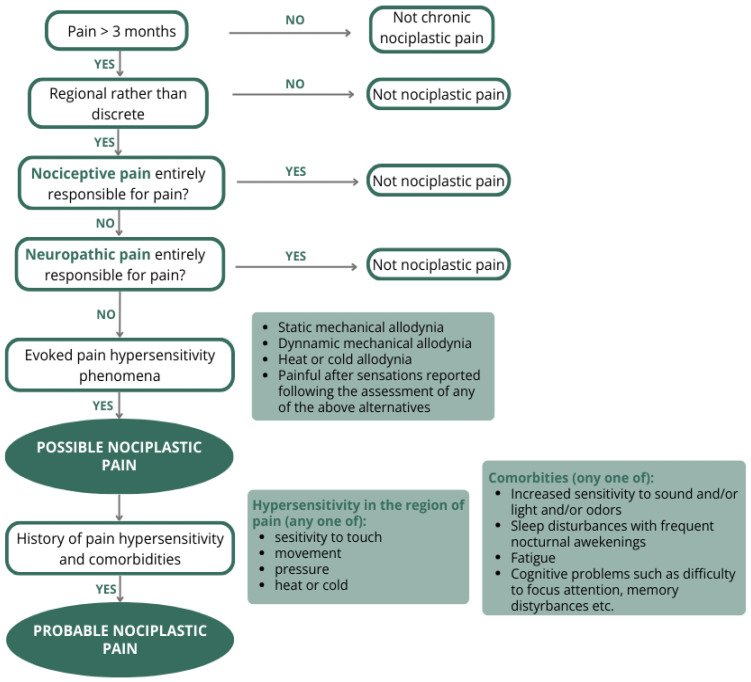
Flowchart for identifying and grading nociplastic pain affecting the musculoskeletal system, adapted from Kosek et al. [10]. The seven clinical criteria proposed by the IASP are presented as sequential steps (Step 1–Step 7), with each step corresponding to one criterion and representing a stage in the clinical evaluation process for identifying nociplastic pain.

In the second stage, we conducted a narrative review with systematic search procedures to determine the applicability of each of the seven clinical criteria proposed in the IASP nociplastic pain criteria [10] (Figure 2) for phenotyping musculoskeletal pain in individuals with DM.

The search was performed in the PubMed and Web of Science databases, including studies from inception to December 2025, with no restrictions on publication date. The search strategy involved the use of specific MeSH terms, such as “diabetes mellitus,” “diabetes complications,” “diabetic neuropathies,” and “diabetic foot”, to address the aspect of DM, as well as terms related to chronic pain, including “musculoskeletal pain,” “chronic pain,” “central nervous system sensitization,” “paresthesia,” “arthralgia,” “back pain,” and “neck pain.” Boolean operators (AND, OR, NOT) were applied to enhance search sensitivity. Full electronic search strategies, including all Boolean equations, are provided in the Supplementary Materials (Table S1). The grey literature was not considered.

Supplementary articles were identified through manual screening of reference lists and citation tracking of the included studies. These studies were assessed for eligibility, and data were extracted using the same criteria applied to all other articles.

#### Eligibility Criteria

No restrictions were applied regarding study design, as the objective was to capture evidence relevant to the mechanisms and clinical features associated with nociceptive, neuropathic, and nociplastic pain in this population. Accordingly, observational studies, interventional clinical studies, and relevant reviews were considered if they provided information pertinent to the proposed framework. Only studies involving human participants and published in English were included. Articles were excluded if they were clearly unrelated to musculoskeletal pain in DM or focused primarily on animal models, specific pharmacological treatments (e.g., pregabalin or gabapentin), or medical conditions irrelevant to musculoskeletal pain (e.g., cardiovascular diseases).

### 2.4. Data Extraction and Synthesis

Two reviewers (LBN and JKF) independently assessed all identified studies by title to exclude those clearly unrelated to our purpose. The abstracts of the selected titles were then analyzed, and the full texts of potentially relevant articles were retrieved for final review. Through a comprehensive reading, evidence pertaining to each of the seven clinical criteria for nociplastic pain proposed by the IASP was identified. These findings were then summarized in an Excel spreadsheet. The extracted data were then collectively interpreted to identify patterns related to the seven IASP nociplastic pain criteria. Evidence was summarized narratively, linking each study’s findings to the corresponding criteria.

## 3. Results

### 3.1. Search Results

**Figure 3:**
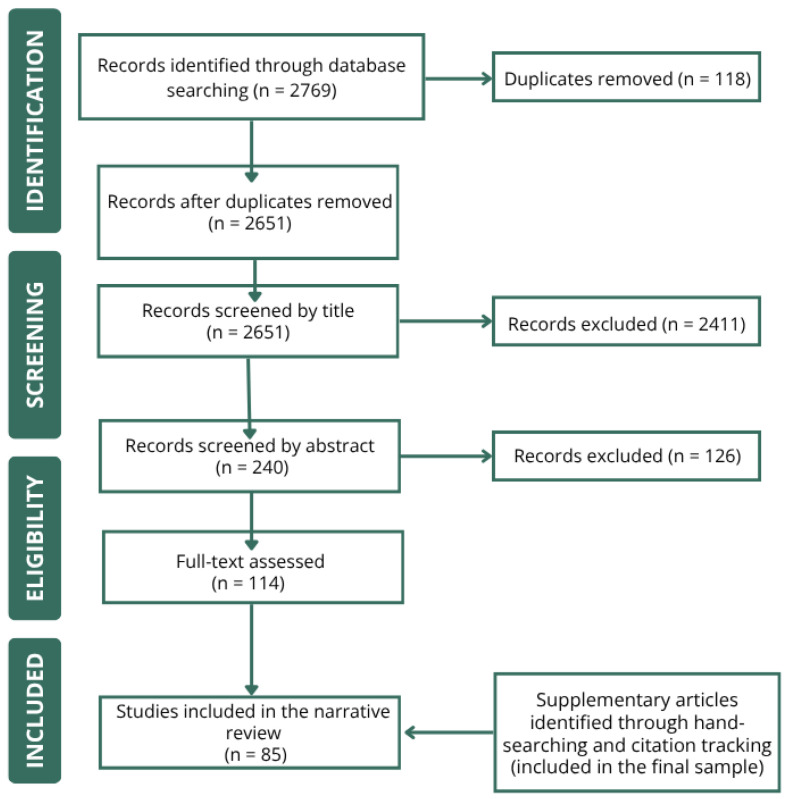
Flow diagram illustrating the study selection process for this narrative review with systematic search procedures, including identification, screening, eligibility, and inclusion of studies.

The electronic search returned 2769 studies. Of these, 118 duplicated records were removed. Subsequently, the titles of the remaining articles were analyzed, resulting in 240 articles selected for a more in-depth review of the abstracts. Following this step, 114 articles were identified as potential candidates for full-text reading. Finally, after a detailed analysis of the full texts, 85 articles were considered significant contributors, providing relevant information for at least one of the seven clinical criteria proposed by the IASP for identifying nociplastic pain. A flow diagram summarizing the study selection process is presented in Figure 3.

The included literature reflects a heterogeneous evidence base. Many studies focus on diabetic neuropathy, particularly painful diabetic polyneuropathy, which is one of the most frequently investigated pain conditions in DM. However, a considerable number of publications also address other musculoskeletal pain conditions, including low back pain, shoulder disorders, osteoarthritis, and generalized musculoskeletal pain. The majority of the literature is observational in nature, with cross-sectional designs predominating, while narrative reviews are also common, reflecting ongoing efforts to synthesize emerging evidence on pain in diabetes. In contrast, comparatively fewer studies investigate the biological and neurophysiological mechanisms underlying pain. Geographically, the literature is largely concentrated in Europe and North America, although contributions from Asia, the Middle East, Africa and South America are also represented.

### 3.2. Evidence and Limitations for Applying the 2021 IASP Criteria for Nociplastic Pain and the Established Clinical Criteria for Neuropathic Pain to People with DM and Musculoskeletal Pain

In this section, the available evidence related to each of the seven clinical criteria proposed by the IASP for nociplastic pain is presented and critically interpreted. Given that this review aims to evaluate the applicability of each criterion to musculoskeletal pain in individuals with DM, the presentation of evidence and its interpretative discussion are integrated within each step, rather than being presented in a separate discussion section.

- Step 1—Pain duration

The identified studies indicate a consistent association between DM and chronic musculoskeletal pain. Epidemiological data suggest that musculoskeletal pain is common among individuals with DM, with chronic pain lasting ≥3 months reported in 49.5% of patients with type 2 DM in a primary care setting in Hong Kong [12]. Several studies included in this review define chronic pain as persisting for ≥3 months in people with DM [5,37,38,39,40,41,42,43,44,45,46,47], in line with international standards, including the IASP criteria for pain phenotyping [10]. Abaraogu et al. [41] identified chronic musculoskeletal symptoms, such as pain and/or stiffness lasting for three months or longer, with chronic low back pain reported in 49.7% of participants with T2DM, compared to 38.9% of those without DM. Similarly, studies such as those by Hassoon et al. [40] and Liberman et al. [46] considered chronic low back pain in people with DM as persisting for ≥3 months to fit into the category of chronic pain. Additionally, there is consistent evidence that the duration of chronic pain in people with DM can be substantial, with reports of pain lasting more than one year [46].

This suggests that chronic musculoskeletal pain in patients with DM often meets the ≥3-month duration criterion proposed in the first step of the IASP classification of nociplastic pain [10]. Importantly, a distinction should be made between chronic musculoskeletal pain and chronic painful diabetic neuropathy. Painful diabetic neuropathy is also considered chronic when pain persists for ≥3 months, but it is defined as pain resulting from abnormalities in the peripheral somatosensory system in individuals with DM [15]. Thus, although both conditions meet the temporal criterion for chronic pain, they represent different underlying mechanisms. Nevertheless, musculoskeletal pain and neuropathic mechanisms may coexist in people with DM, and their potential interaction will be explored in the following steps.

- Step 2—Pain distribution (regional rather than discrete)

The identified studies suggest that people with DM report more widespread pain, including regions above and below the waist, compared to individuals without DM [39,42]. Furthermore, there may also be an association between DM and chronic widespread pain, characterized by pain lasting ≥3 months and involving four or more body sites [43,44]. Other studies observed that a significant proportion of people with DM and neuropathy frequently report pain in two or more locations beyond the lower limbs [48] or suffer from other chronic musculoskeletal pain conditions [49]. People with painful neuropathy are also more likely to experience pain in various body locations [49]. Importantly, factors such as obesity, waist circumference, depression, and neuropathy severity have been associated with an increased risk of painful diabetic neuropathy [50,51] and may act as potential confounders influencing pain distribution.

The findings from this step are consistent with a Delphi study in which 82% of the 49 experts agreed that diffuse, widespread, or poorly localized pain is a distinctive characteristic of nociplastic pain [52]. However, the presence of widespread pain is supportive of, but not sufficient for, the diagnosis of nociplastic pain, and should be interpreted alongside other clinical features. This highlights the need to consider the predominant underlying pain mechanisms.

- Step 3—Nociceptive pain mainly responsible for pain

The identified studies indicate that musculoskeletal disorders associated with DM may have a predominantly nociceptive origin. Several studies report a high prevalence of painful conditions with nociceptive characteristics in people with DM, such as frozen shoulder, rotator cuff disorders, intervertebral disc degeneration, osteoarthritis, and limited joint mobility syndrome [53,54,55,56,57,58,59,60]. These conditions appear to be partly related to hyperglycemia, which increases advanced glycation end-product (AGE) levels and affects collagen structure, leading to alterations in strength, stability, and organization of tendons, ligaments, and cartilage [61].

However, epidemiological studies have shown that abnormalities like intervertebral disc degeneration (IVDD) [62], degenerative cartilage tears in the knee [63,64], and tendon tears in the shoulder [65] are also common in asymptomatic individuals, indicating that structural alterations may not entirely explain pain experiences. This dissociation is evident even in people with DM, as ultrasonographic arthritic knee changes are highly prevalent in individuals with type 2 DM both with and without knee pain, while specific local nociceptive diagnoses are identified in only a small proportion of individuals [64]. Notably, the presence of structural abnormalities does not always result in pain, illustrating a structure–symptom mismatch that may contribute to overmedicalization in people with DM, as interventions could be guided primarily by imaging findings rather than patient-reported symptoms. Furthermore, evidence suggests that pain in people with DM is not exclusively nociceptive, as individuals often report chronic pain in multiple body regions [66,67,68]. The assessment of neuropathic and nociplastic features is frequently overlooked, despite IASP recommendations for comprehensive pain phenotyping [10,69].

Taken together, these findings suggest that although musculoskeletal disorders associated with DM may imply the presence of nociceptive drivers, their presence does not guarantee the predominance of nociceptive pain. Indeed, the presence of nociceptive drivers does not rule out contributions from nociplastic and neuropathic mechanisms. In this context, mixed musculoskeletal conditions involving both nociceptive and neuropathic components have already been described in people with DM [70,71], reinforcing the complexity of pain in this population and the need for a multidimensional approach to its management. This perspective is consistent with IASP recommendations for pain phenotyping [10], which recognize that mixed pain presentations are common in other pain conditions [31] beyond the DM population.

- Step 4—Neuropathic pain mainly responsible for pain

An extensive range of studies investigating diabetic neuropathy is available since it is a very common complication of DM. Peripheral symmetric polyneuropathy emerges as the most common form of manifestation, which may or may not be accompanied by pain. A high prevalence of neuropathic pain among individuals with diabetic neuropathy has been reported, ranging from 20.3 to 63.9% [37,48,72,73,74,75]. The great discrepancy in epidemiological data may be attributed to the use of different diagnostic criteria for identifying neuropathic pain among the studies. When considering only studies that assessed neuropathic pain in people with DM by means of the IASP criteria [69], the prevalence ranges from 37% to 42% [48,74].

Surprisingly, there has been an increase in studies adopting the clinical criteria proposed by the IASP [71,76]. Other painful conditions in people with DM involve neuropathic mechanisms distinct from classic distal polyneuropathy, such as carpal tunnel syndrome [77], neuropathic arthritis or Charcot joints [36], diabetic amyotrophy [78], and reflex sympathetic dystrophy [46,79]. These conditions arise from nerve involvement but can also lead to structural changes in musculoskeletal tissues, producing pain features that may overlap with nociceptive mechanisms. Neuropathic pain in IVDD and shoulder disorders similarly involves mechanisms distinct from classic distal polyneuropathy. Jin et al. [56] found that type 2 DM has a causal effect on IVDD, regardless of body mass index, and people with type 2 DM show a 6.9% increased risk of developing IVDD compared to those without DM. The pathophysiological changes in the cartilaginous endplates of the discs in IVDD may increase the propensity for disc protrusions, which, in turn, can contribute to nerve root compression and contribute to the development of low back pain with possible nociceptive or neuropathic characteristics. Likewise, Alabdali et al. [54] investigated the prevalence of neuropathic shoulder pain in people with type 2 DM and found that pain was present in 3% of patients when adopting a score of 5 or more in the Douleur Neuropatique 4 (DN4) screening instrument, used as a diagnostic criterion for neuropathic pain. Beyond identifying the predominant pain mechanisms, it is also relevant to explore features of altered pain processing, such as hypersensitivity phenomena.

- Step 5—Pain hypersensitivity phenomena

This step involves screening for pain hypersensitivity in the pain region by means of static or dynamic mechanical allodynia, heat or cold allodynia, and painful after-sensations reported following these assessments [10]. Sensory phenotyping allows distinguishing between sensory loss (negative sensory signs, e.g., reduced perception to touch or temperature) and gain-of-function phenomena (positive sensory signs, e.g., mechanical or thermal allodynia and hyperalgesia) [69,76]. These patterns reflect different underlying pathophysiological mechanisms and may inform targeted assessment and management.

In people with DM and musculoskeletal pain, there remains a significant gap in the literature regarding the investigation of evoked pain hypersensitivity phenomena. Existing studies have primarily focused on the evaluation of sensory profiles in people with diabetic neuropathy, whether painful or not. Akintoye et al. [80] found that people with painful diabetic neuropathy have significantly reduced pain thresholds and beta-endorphin levels compared to individuals without diabetic neuropathy and to healthy controls. However, this study did not adopt the IASP criteria for identifying neuropathic pain [69]. Additionally, Granovsky et al. [81] compared the efficiency of endogenous pain modulation in people with painful diabetic neuropathy (assessed by IASP criteria) with those with non-painful diabetic neuropathy and did not find significant differences in pressure pain modulation between groups; however, individuals with painful neuropathy showed more efficient heat pain modulation. Nevertheless, the study by Themistocleous et al. [76] revealed that dynamic mechanical allodynia, specifically evoked by brushing, was the only “gain-of-function” phenomenon that differentiated participants with painful diabetic neuropathy from those with non-painful diabetic neuropathy. This phenomenon was observed in 15% of individuals with painful diabetic neuropathy, indicating a possible dysfunction in central processing of sensory information in these individuals [76].

However, since there is limited evidence on pain hypersensitivity assessment in other DM-related painful musculoskeletal conditions (beyond diabetic neuropathy), and the available evidence in people with painful diabetic neuropathy is inconsistent, it cannot be concluded that people with DM truly present alterations related to “sensory gain of function” [76,82,83]. Moreover, most studies use assessments reflecting central alterations in sensory processing, such as temporal summation, conditioned pain modulation, or exercise-induced hypoalgesia, and the reliability of these tests in clinical settings is still uncertain [10]. A key distinction is that peripheral sensitization involves increased excitability of nociceptors in the affected region, while central sensitization reflects amplified pain processing within the central nervous system. Both mechanisms can lead to pain hypersensitivity, although through different processes and with distinct clinical implications; in particular, central sensitization is one of the mechanisms often associated with nociplastic pain [24,84]. For this reason, further research is warranted to explore hypersensitivity phenomena in people with DM and painful musculoskeletal conditions. Additionally, assessing the history of hypersensitivity reported by patients can provide complementary clinical information.

- Step 6—History of pain hypersensitivity

This step focuses on evaluating whether people with DM report hypersensitivity in the painful region. Information can be gathered through interviews exploring sensitivity to touch, movement, pressure, or temperature variations. This step also has a lack of evidence to support its application in DM, revealing a significant gap in the literature. Similarly to the previous step, the limited existing evidence seems to be predominantly related to diabetic neuropathy, which is indeed the most prevalent and devastating condition for people with DM.

For instance, Knauf and Koltyn [85] identified that people with painful diabetic neuropathy experienced elevated levels of muscle pain during exercise and lacked exercise-induced hypoalgesia compared to people with DM without painful diabetic neuropathy. The authors hypothesized about a possible movement hypersensitivity in this population. Additionally, several studies [86,87,88,89] examined the validity of the Michigan Neuropathy Screening Instrument (MNSI) as a screening and diagnostic tool for diabetic neuropathy in different populations and clinical contexts. The MNSI consists of two components, a 15-item self-administered questionnaire and a physical examination of the lower limbs, addressing hypersensitivity to touch and movement and hyposensitivity to temperature in the feet and legs of the patients [86]. Despite its primary use in cases of diabetic neuropathy, this tool includes items that may reveal the potential presence of hypersensitivity symptoms among individuals with DM. However, it should be noted that the MNSI was not designed to detect nociplastic pain, highlighting a methodological mismatch when assessing central or widespread hypersensitivity.

Evidence supporting the application of this step for individuals with DM with musculoskeletal pain is still limited, and it would be interesting for further studies to investigate self-reported hypersensitivity to better understand its presentation and clinical impact across a broader range of patients. Such information can provide complementary clinical insight and may contribute to the evaluation of comorbid symptoms in the following step.

- Step 7—Comorbid symptoms

The last step implies screening for comorbid symptoms, including fatigue, difficulties with cognition such as attention and memory issues, sleep problems, and heightened sensitivity to sensory inputs like light, sound, or odors. In people with DM and musculoskeletal pain, some evidence on the presence of cognitive dysfunction and sleep disorders stands out. Croosu et al. [79] found that individuals with type 1 DM performed worse on cognitive tests, especially in the areas of memory and language, when compared to healthy controls. However, in a subgroup analysis, no difference in cognitive function was observed among individuals with type 1 DM when stratified according to pain status and neuropathy classification (painful, painless, or absent diabetic neuropathy). A study performed by Nunley et al. [90] observed a fivefold higher prevalence of significant cognitive impairment in middle-aged adults with type 1 DM than in healthy controls (28% vs. 5%), independent of education and age, but they did not consider the presence or experience of pain in the sample. Additionally, two studies [91,92] did not identify differences in cognitive function (by means of the Test Your Memory instrument) between type 2 DM patients with and without diabetic neuropathic pain, but one of them [91] observed that obesity and a greater disease duration were factors related to a greater risk of cognitive impairment in people with diabetic neuropathic pain.

Different studies [92,93,94,95] reported high rates of sleep disturbances in people with painful diabetic neuropathy. In addition, individuals with painful diabetic neuropathy presented with worse sleep rates when compared to individuals with painless diabetic neuropathy [92], the general population [94], and people with other chronic diseases [94]. Mehrdad et al. [96] found significant subjective sleep disturbances in people with DM, both types 1 and 2, and identified that one of the most reported factors of sleep interruptions was pain. Furthermore, poor sleep quality was linked to higher HbA1c and 2HPPBS and poor glycemic control [96]. Additionally, one study [97] observed a higher prevalence of chronic fatigue in individuals with type 1 DM relative to individuals without DM, and the condition was linked to factors such as age, depressive symptoms, pain, sleep disturbances, reduced self-efficacy related to fatigue, and physical inactivity.

Within the context of step 7, a key limitation of the current literature is that most of the studies considered only the condition of painful diabetic neuropathy and did not assess comorbid symptoms in the presence of other painful musculoskeletal conditions related to DM. This limits our understanding of how comorbid symptoms may relate to different musculoskeletal pain presentations and the potential underlying mechanisms in people with DM. In addition, there were no studies that assessed comorbid symptoms including heightened sensitivity to auditory, visual, and olfactory stimuli in people with DM and musculoskeletal pain. Given these gaps, approaches used in other chronic pain conditions associated with central sensitization, such as fibromyalgia, may provide useful insights. In these conditions, cluster and factor analyses have identified subgroups with distinct physical, cognitive, and psychological symptom profiles [98,99]. Applying a similar approach to DM-related musculoskeletal pain could help reveal symptom patterns and underlying nociplastic mechanisms, ultimately supporting more comprehensive and targeted assessment and management.

## 4. Limitations

This review has some limitations that deserve consideration. One limitation is that, although the study was supported by systematic search procedures, it was conducted as a narrative review instead of a formal systematic review, which may limit the extent and reproducibility of the search process. Another limitation is related to the applicability of the IASP nociplastic pain criteria to musculoskeletal pain in individuals with DM, which was evaluated through interpretation of the available literature, since most studies were not originally designed to assess these criteria. In addition, the included evidence shows heterogeneity regarding study designs, populations, and pain conditions, while differences in the definitions and assessment methods of musculoskeletal pain across studies may further limit comparability of findings. Furthermore, much of the literature focuses primarily on painful diabetic neuropathy rather than other musculoskeletal pain conditions related to DM. Despite these limitations, this review provides a structured synthesis of the available evidence and highlights important gaps for future research.

## 5. Conclusions

The available literature indicates that musculoskeletal pain in individuals with DM has not yet been systematically investigated using the clinical criteria proposed by the IASP for pain phenotyping. While nociceptive and neuropathic pain mechanisms are well documented in this population, evidence related to central sensitization and nociplastic pain remains scarce. This gap limits a comprehensive, mechanism-based understanding of pain in people with DM and may contribute to the continued use of generalized management approaches instead of mechanism-based interventions.

Future research should prioritize longitudinal cohort studies that track pain duration and distribution while incorporating approaches such as quantitative sensory testing and multidimensional symptom assessment to better characterize pain mechanisms and identify clinically meaningful subgroups. In addition, the development of standardized pain phenotyping frameworks could help integrate these assessments and support more precise, mechanism-based classification and individualized management strategies for people with DM. Such advances would also have important clinical implications, as more accurate phenotyping may help reduce misclassification and guide more appropriate treatment selection, for example, by avoiding over-reliance on pharmacological approaches in favor of multimodal, mechanism-based care.

## Data Availability

Not applicable.

## Supplementary Materials

The following supporting information can be downloaded at https://www.mdpi.com/article/10.3390/jcm15072639/s1: Table S1: Complete search strategies and Boolean equations used in the systematic search.
