# Supplementary material for: Beyond Neuropathy: The Mechanisms and Phenotypes of Diabetes-Related Musculoskeletal Pain
Source: J Clin Med. 2026 Mar 31;15(7):2639. doi: 10.3390/jcm15072639 (PMC13073196; doi:10.3390/jcm15072639)
Supplement: Supplementary file 1 [file jcm-15-02639-s001.zip › jcm-4175623-supplementary.pdf]

Supplementary material

Table S1: Complete search strategies and Boolean equations used in the systematic search

Database	Search strategy	Filters	Results
PubMed	(("diabetes mellitus"[MeSH Major Topic] OR "diabetes complications"[MeSH Terms] OR "diabetic neuropathies"[MeSH Terms] OR "diabetic foot"[MeSH Terms]) AND ("musculoskeletal pain"[MeSH Terms] OR "chronic pain"[MeSH Terms] OR "central nervous system sensitization"[MeSH Terms] OR "paresthesia"[MeSH Terms] OR "arthralgia"[MeSH Terms] OR "back pain"[MeSH Terms] OR "neck pain"[MeSH Terms])) NOT ("animal experimentation"[MeSH Terms] OR "rats"[MeSH Terms] OR "mice"[MeSH Terms] OR "pregabalin"[MeSH Terms] OR "gabapentin"[MeSH Terms] OR "cardiovascular diseases"[MeSH Terms] OR "diabetes, gestational"[MeSH Terms])	Humans	394
Web of Science	TS=(diabetes mellitus OR diabetes mellitus type 1 OR diabetes complications OR diabetic neuropathies OR diabetic foot) AND TS=(musculoskeletal pain OR chronic pain OR central nervous system sensitization OR paresthesia OR shoulder pain OR back pain OR neck pain) NOT TS=(rats OR mice OR pregabalin OR gabapentin OR gestational diabetes OR chest pain)	Document types: Article, Review Article, Early Access	2,375
